# Polymorphisms of *Plasmodium falciparum* k13-propeller gene among migrant workers returning to Henan Province, China from Africa

**DOI:** 10.1186/s12879-017-2634-z

**Published:** 2017-08-10

**Authors:** Chengyun Yang, Hongwei Zhang, Ruimin Zhou, Dan Qian, Ying Liu, Yuling Zhao, Suhua Li, Bianli Xu

**Affiliations:** Department of Parasite Disease Control and Prevention, Henan Province Center for Disease Control and Prevention, Zhengzhou, 450016 People’s Republic of China

**Keywords:** Imported malaria, *Plasmodium falciparum*, K13-propeller gene, Artemisinin resistance, Africa, Henan Province

## Abstract

**Background:**

Henan Province has been in the malaria elimination stage, with all reports of the disease being imported since 2012 and over 90% coming from Africa. Surveillance and population studies are essential for the early detection and subsequent prevention of the spread of drug resistance. The K13-propeller gene was recently identified as a proposed molecular marker of artemisinin (ART) resistance. In this study, we detected mutations of the K13-propeller gene in samples taken from imported malaria cases in Henan Province from 2012 to 2015.

**Methods:**

There were 483 samples that were obtained from *Plasmodium falciparum*–infected malaria migrant workers who returned to Henan Province from Africa between 2012 and 2015. The single nucleotide polymorphisms in the K13-propeller gene were assessed by nested PCR with DNA sequencing. Frequency and geographic difference of K13-propeller gene mutant types were analyzed.

**Results:**

Of 483 patients, 476 were cured and 7 died. There were no K13-propeller mutations in the blood samples from the 7 patients who died, but there were 23 different genotypes of the K13-propeller that were observed in 24 (4.97%) of the samples. C580Y, which was the predominant one in the resistance of ART, was not detected in the samples, but R539T and P574L which have also been associated with ART resistance, were observed in two samples from Angola and Equatorial Guinea. No mutations were detected in 11 samples from North Africa. The frequency of the K13-propeller was 6.50% (8/123) in Central Africa, followed by East Africa (1/19, 5.26%), West Africa (9/198, 4.55%) and South Africa (6/132, 4.55%). There was no significant difference among these four areas (*P* = 0.795).

**Conclusion:**

R539T and P574L were found in migrant workers who traveled from Africa to Henan Province, although the frequency of the K13-propeller mutants was low. These data may enrich the molecular surveillance of antimalarial resistance and will be helpful for developing and updating the antimalarial policy in Henan Province.

## Background

Malaria is the most critical human parasitic disease caused by Plasmodium, it is a serious threat to human health, and there were about 214 million cases of malaria and 438,000 deaths from it in 2015, of which 90% occurred in Africa [1]. In China, an action plan for malaria elimination was launched in 2010, and the Henan government carried out the action the same year. Indigenous *P. falciparum* was successfully eliminated in 1988 and no indigenous malaria cases have been reported in Henan Province since 2012 [2]. However, imported malaria cases have increased year after year. *P. falciparum* which was imported from Africa, has been mainly responsible for the increasing impacts [3, 4].

The effectiveness of antimalarial medicines is a powerful guarantee for malaria control and elimination. Artemisinin-based combination therapies (ACTs) were recommended as the first-line treatment against uncomplicated *P. falciparum* infection in 2006 by the World Health Organization (WHO) [5]. Artemisinin has proven to be the most successful antimalarial drug over 10 years, and the morbidity and mortality of malaria have fallen dramatically due to the use of ACT in highly endemic areas of the world [6]. However, as with other drugs, the curative effect of ACT has declined gradually along with its use. The emergence of *P. falciparum* resistance to artemisinin and its derivatives was first reported in 2008 in Western Cambodia [7]. In 2009, Dondorp et al. [8] also reported that the susceptibility of *P. falciparum* in vivo to ACT was reduced. Then, ACT resistance became prevalent in the Greater Mekong Subregion (GMS) [9–11]. K13-propeller, as a molecular marker of artemisinin-resistance, was identified by Ariey in 2014 [12]. Since then, numerous studies have been conducted about K13-propeller mutations. The K13-propeller mutations associated with artemisinin-resistance were mainly found in Southeast Asia, with C580Y being the predominant one. At the same time, many studies were carried out in Africa. While few resistance-associated mutations have been found in Africa [13], there have been numerous mutations found in the K13-propeller. In 2015, Feng et al. [14] reported that C580Y and R539T were observed, and the most prevalent mutation was C580Y, with the rate of 2.7% in the samples from Ghana.

As a proposed molecular marker of ART resistance, the K13-propeller was recently identified and has been studied in an increasing number of reports. Mutations in the K13-propeller were first reported in 2008 in western Cambodia [11]. More mutations in this gene have been also subsequently found in resistant parasites in samples from Vietnam and Burma. To date, more than 200 nonsynonymous mutations in the K13-propeller gene have been reported, of which mutations of N458Y, Y493H, R539T, I543T, R561H, and C580Y were validated to be associated with ART resistance, and M476I and M579I were also reported to be associated with resistance in vivo or in vitro tests [15, 16]. In this study, we evaluated the prevalence of polymorphisms in the K13-propeller gene from imported *P. falciparum* patients who returned from Africa to Henan Province during 2012- 2015.

## Methods

### Sample collection and DNA extraction

Blood samples were obtained from migrant workers with *P. falciparum* infection prior to treatment. All of the patients returned from Africa to Henan Province between 2012 and 2015. Thick and thin blood films were smeared and stained with 10% Giemsa for 20 min. The final diagnosis was confirmed by microscopic examination of Giemsa-stained thick blood films and nested PCR. For each patient, approximately 200 μl of finger-prick blood was spotted on Whatman No. 903 filter paper. All of the blood samples were labeled with study numbers, names, and dates and stored in individual plastic bags at −20 °C until use. DNA was extracted from the blood samples using the QIAamp DNA Mini kit (QIAGEN Inc., Germany) according to the manufacturer’s instructions and stored at −20 °C for use in PCR assays.

### K13-propeller gene amplification and sequencing

The K13-propeller gene was amplified by the nested PCR method as previously described [17]. Nested PCR was performed to amplify the fragments of the K13-propeller using a DNA thermal cycler (Mastercycler nexus, Eppendorf Ltd., Germany). The first-round PCR was performed in a 25-μl reaction volume containing 8.5 μl of ddH_2_O, 1.0 μl each of the primers Artinner F (GCCTTGTTGAAAGAAGCAGAA) and Artouter R (CGCCATTTTCTCCTCCTGTA) (10 μmol/l), 12.5 μl of 2 × Go Taq Green Master Mix (Promega, USA), and 3.0 μl of DNA template. The PCR reaction condition was 95 °C for 15 min, followed by 35 cycles at 94 °C for 1 min, 58 °C for 1 min, and 72 °C for 2 min, and a final extension at 72 °C for 5 min. The second round contained the primers ArtinnerF and ArtinnerR (GTGGCAGCTCCAAAATTCAT) and 5 μL of the first-round product as the template, with the same cycling conditions as the first round. The amplified products were sequenced by Sangon Biotech Co. Ltd. (Shanghai, China). The primers were also synthesized by Sangon Biotech Co. Ltd.

### Sequencing alignments and data analysis

Sequences were analyzed with the BLAST program. Sequences were aligned to reference PF3D7_1343700 (http://www.plasmodb.org) using the BioEdit Sequence Alignment Editor. The K13-propeller allele frequency was calculated with Microsoft Excel to assess the differences by SPSS 17.0. Pearson’s chi-square test was used to determine the significance of the results. *P* values were calculated and considered to be statistically significant at <0.05.

## Results

### Epidemiological and clinical characteristics of patients

A total of 483 samples were collected from migrant workers who had returned from 27 countries in Africa and were diagnosed with *P. falciparum* in Henan Province during 2012-2015. Of them, 89 were collected in 2012, 125 were obtained in 2013, 141 were taken in 2014, and 128 were collected in 2015. Of the 483 patients, the majority (40.99%, 198/483) had returned from West Africa, followed by South Africa (27.33%, 132/483) and Central Africa (25.47%, 123/483). Only a minority of the patients came back from East Africa (3.93%, 19/483) and North Africa (2.28%, 11/483) (Table 1). The male: female ratio was 79.5:1 (477/6), and the age range was 17 to 70 with the mean age of 37.76 ± 9.30 years.

**Table 1:** Geographic origin, year of sample collection, and distribution of K13-propeller polymorphisms

Region	Country	Year of collection				Total	Mutation N (%)	Year of mutation detected (n)
		2012	2013	2014	2015			
North Africa		3	5	1	2	11	0 (0.00%)	
	Sudan	3	3	1	2	9	0 (0.00%)	
	Libya	0	2	0	0	2	0 (0.00%)	
South Africa		19	45	33	35	132	6 (4.55%)	2013 (1), 2014 (3), 2015 (2)
	Mozambique	6	1	2	1	10	0 (0.00%)	
	Zambia	1	7	7	4	19	0 (0.00%)	
	Angola	12	37	23	30	102	5 (4.90%)	2013 (1), 2014 (2), 2015 (2)
	Zimbabwe	0	0	1	0	1	1 (100.00%)	2014 (1)
East Africa		0	2	7	10	19	1 (5.26%)	2015 (1)
	Ethiopia	0	0	1	1	2	0 (0.00%)	
	Kenya	0	1	2	1	4	0 (0.00%)	
	Uganda	0	1	1	2	4	1 (25.00%)	2015 (1)
	Tanzania	0	0	3	6	9	0 (0.00%)	
West Africa		45	43	60	50	198	9 (4.55%)	2012 (2), 2013 (1), 2014 (5), 2015 (1)
	Burkina Faso	0	0	0	1	1	0 (0.00%)	
	Mali	0	1	3	0	4	0 (0.00%)	
	Togo	0	0	5	0	5	0 (0.00%)	
	Benin	0	1	4	0	5	1 (20.00%)	2014 (1)
	Ivory Coast	1	0	3	4	8	0 (0.00%)	
	Liberia	5	9	4	4	22	2 (9.09%)	2012 (1), 2013 (1)
	Ghana	3	7	6	7	23	1 (4.35%)	2012 (1)
	Sierra Leone	7	6	5	6	24	1 (4.17%)	2014 (1)
	Guinea	9	7	3	15	34	1 (2.94%)	2015 (1)
	Nigeria	20	12	27	13	72	3 (4.17%)	2014 (3)
Central Africa		22	30	40	31	123	8 (6.50%)	2013 (2), 2014 (5), 2015 (1)
	Central African Republic	3	0	0	0	3	0 (0.00%)	
	Chad	0	1	4	1	6	0 (0.00%)	
	Gabon	2	2	2	0	6	0 (0.00%)	
	Congo, DRC	0	0	3	4	7	1 (14.29%)	2014 (1)
	Congo	3	2	4	5	14	0 (0.00%)	
	Cameroon	0	5	13	4	22	0 (0.00%)	
	Equatorial Guinea	14	20	14	17	65	7 (10.77%)	2013 (2), 2014 (4), 2015 (1)
Total		89	125	141	128	483	24(4.97%)	2012 (2), 2013 (4), 2014 (13), 2015 (5)

The clinical characteristics, including fever, chills, sweating, headache, and diarrhea, were analyzed. There were 99.79% of the patients who had a fever and 21.95% (106/483) who had serious complications, including cerebral lesion, coma, and hemolysis (see Table 2).

**Table 2:** Clinical characteristics of the patients

Symptom	No. of cases *n* = 483	%
Fever	482	99.79
Chills	334	69.15
Sweating	293	60.66
Diarrhea	51	10.56
Headache	260	53.83
Cerebral lesion	33	6.83
Severe anemia	11	2.28
Liver and kidney damage	38	7.87
Hemolysis	2	0.41
Coma	5	1.04
Gastrointestinal damage	8	1.66
Other symptoms	9	1.86

After diagnosis, the patients with no complications were treated with Dihydroartemisinin and Piperaquine phosphate tablets, while the others were treated with injected Artesunate at the same time for symptomatic treatment. The results of the treatment were that most patients recovered, with the exception of the 7 patients who died; K13-propeller mutations were not observed in the samples from these 7 patients.

### K13-propeller point mutations

A 751-bp fragment was amplified by nested PCR for each of the 483 samples. All of the products were sequenced successfully, and 4.97% (24/483) contained single nucleotide polymorphisms (SNPs) at 23 locations, of which 8 were described previously and 15 were unreported (Table 3). Notably, the R539T, and P574L mutations that were associated with ART resistance were observed in the samples from Angola and Equatorial Guinea. The M476I substitution was also present in one sample from Equatorial Guinea. The M476I mutation which was produced with artemisinin tolerance in vitro in a Tanzanian strain, was developed specifically to test ACT activity in a test tube. However, none of the I543T, Y493H, and C580Y mutations, which were reported to be directly associated with ART resistance, were observed in these samples.

**Table 3:** Polymorphisms observed in the K13-propeller in 11 countries

Country(n)	Codon position.	Amino acid reference	Nucleotide reference	Amino acid mutation	Nucleotide mutation	Prevalence of mutation (%)	Year (n)
Angola (102)						4.90(*n* = 5)	
	471^a^	R	CGT	R	CGC	1.96 (*n* = 2)	2013 (1), 2015 (1)
	539^b^	R	AGA	T	ACA	0.98 (*n* = 1)	2014 (1)
	579	M	ATG	I	ATT	0.98 (*n* = 1)	2015 (1)
	613	Q	CAA	E	GAA	0.98 (*n* = 1)	2014 (1)
Zimbabwe(1)						100(*n* = 1)	
	664	N	AAT	D	GAT	100(*n* = 1)	2014 (1)
Uganda(4)						25(*n* = 1)	
	578	A	GCT	S	TCT	25(*n* = 1)	2015 (1)
Benin(5)						20(*n* = 1)	
	662	F	TTT	C	TGT	20(*n* = 1)^1^	2014 (1)
	664	N	AAT	D	GAT	20(*n* = 1)^1^	
Liberia(22)						9.09(*n* = 2)	
	629	N	AAT	S	AGT	4.55(*n* = 1)	2013 (1)
	464	D	GAT	N	AAT	4.55(*n* = 1)	2012 (1)
Ghana(23)						4.35(*n* = 1)	
	648	D	GAT	N	AAT	4.35(*n* = 1)^2^	2012 (1)
	664	N	AAT	D	GAT	4.35(*n* = 1)^2^	
Sierra Leone(24)						4.17(*n* = 1)	
	556	E	GAA	K	AAA	4.17(*n* = 1)^3^	2014 (1)
	648	D	GAT	Y	TAT	4.17(*n* = 1)^3^	
Guinea(34)						2.94(*n* = 1)	
	578	A	GCT	S	TCT	2.94 (*n* = 1)	2015 (1)
Nigeria(72)						4.17(*n* = 3)	
	496^a^	G	GGT	G	GGC	0.14(*n* = 1)	2014 (1)
	610	K	AAA	R	AGA	0.14 (*n* = 1)	2014 (1)
	626	A	GCA	T	ACA	0.14(*n* = 1)^4^	2014 (1)
	627^a^	A	GCT	A	GCA	0.14(*n* = 1)^4^	
Congo, DRC(7)						14.29(*n* = 1)	
	613	Q	CAA	E	GAA	14.29(*n* = 1)	2014 (1)
Equatorial Guinea(65)						10.77(*n* = 7)	
	464	D	GAT	N	AAT	1.54(*n* = 1)	2014 (1)
	476	M	ATG	I	ATA	1.54(*n* = 1)^5^	2013 (1)
	589	V	GTC	I	ATC	1.54(*n* = 1)^5^	
	574^b^	P	CCT	L	CTT	1.54(*n* = 1)	2013 (1)
	578	A	GCT	T	ACT	1.54(*n* = 1)^6^	2014 (1)
	579	M	ATG	I	ATT	1.54(*n* = 1)^6^	
	658	K	AAA	Q	CAA	1.54(*n* = 1)	2014 (1)
	663	L	CTA	V	GTA	1.54(*n* = 1)	2014 (1)
	664^a^	N	AAT	N	AAC	1.54(*n* = 1)	2015 (1)

The same number indicated that the two mutations were found in one sample

^a^Synonymous mutation

^b^Mutations associated with ART resistance

Of the 23 mutations, there were 4 synonymous mutations and 19 nonsynonymous mutations. The mutations occurred in 1-3 clones (0.21-0.62%). Of the nonsynonymous mutations, N664D was the most prevalent (0.62%, *n* = 3), while R471R was the most prevalent (0.41%, *n* = 2) of the synonymous mutations. The rest were found in only one sample. Furthermore, six patients were found to have six mixed genotypes: I_476_I_589_, K_556_Y_648_, T_578_I_579_, T_626_A_627_, N_648_D_664_, and C_662_D_664_ (Table 3).

### Distribution of K13-propeller point mutations

**Fig. 1:**
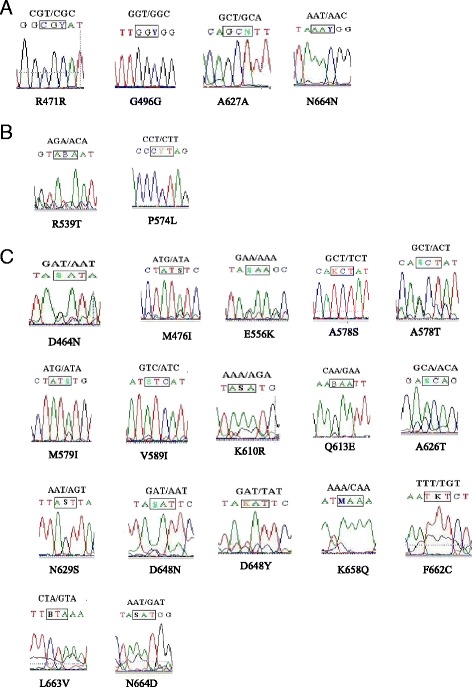
Types of nucleotide sequence mutations detected in Plasmodium falciparum isolates imported from Africa in Henan province. The position of mutations was indicated by a rectangle box. N664D was observed in three samples and R471R, A578S, M579I and Q613E were found in two samples. The rest mutations were detected in only one sample. **a** Four synonymous mutations detected in isolates from Africa; **b** Two nonsynonymous mutations were associated with ART resistance; **c** 17 nonsynonymous mutations weren’t associated with ART resistance.; (B = C/G; K = G/T; M = A/C; N = T/A; S = G/A; Y = T/C)

A total of 23 point mutations were detected in the isolates from 27 countries in Africa during 2012-2015 in Henan Province. The mutations were distributed in 11 countries. There were no mutations detected in 11 isolates from 2 countries in North Africa. Five polymorphisms (R471R, R539T, M579I, Q613E, and N664D) were detected in 132 isolates from 4 countries in South Africa, with 4.55% (6/132) prevalence. There was only one mutation, A578S, which was detected in 19 isolates from four countries in East Africa, with 5.26% (1/19) prevalence. There were 12 polymorphisms (D464N, G496G, E556K, A578S, K610R, A626T, A627A, N629S, D648N, D648Y, F662C, and N664D) that were found in 198 isolates from 10 countries in West Africa, with 4.55% (9/198) prevalence. There were 10 polymorphisms (D464N, M476I, P574L, A578T, M579I, V589I, Q613E, K658Q, L663 V, and N664 N) that were detected in 123 isolates from 7 countries in Central Africa, with 6.50% (8/123) prevalence (see Fig. 1, Tables 1 and 3). There was no significant difference among these 4 areas (*P* = 0.795).

## Discussion

In this study, the sequencing of the K13-propeller gene from 483 *P*. *falciparum* samples collected from Africa identified 23 point mutations in 24 samples. We observed 4 synonymous and 19 nonsynonymous mutations, of which 8 mutations had been reported before and 15 had yet to be described [18–21]. All of the samples were from 27 countries in Africa, and 24 samples with mutations were distributed in 11 countries. The K13-propeller mutations were most prevalent in Equatorial Guinea, Angola, and Nigeria; P574L and R539T were detected in the samples from Equatorial Guinea and Angola. In our samples, we could not detect C580Y in the 23 isolates from Ghana, a possible reason for this might be that the samples from Ghana were small. The results in our study were not consistent with the report by Feng et al. [14] and other reports with no mutations associated with artemisinin resistance in Africa [17, 22], but the low mutation rate agreed with other reports in samples from Africa [23, 24]. The findings in our study indicated that the mutants of the K13-propeller gene alleles exist in migrant workers returning from Africa, which required us to focus on these individuals and strengthen the early monitoring of the K13-propeller.

In this study, the total rate of mutation was 4.97% and the major mutations were from West Africa, South Africa, and Central Africa. The difference of the mutations in these regions was not significant. In the 102 samples from Angola, one K13-propeller resistance-confirmed mutation, R539T was observed in one sample and the mutation rate was 0.98%. We also detected another resistance-confirmed mutation, P574L, in one isolate from Equatorial Guinea and the rate was 1.54% (1/65). The other mutations associated with ART resistance were not found in our study, but the extensive distribution of the K13-propeller mutations and the emergence of the mutations (P574L and R539T) being directly related to ART resistance suggests that there are potential ART-resistant genes in Africa that could make ART-resistant mutations spread all over the world. Routine monitoring must continue in order to ensure that the recommended ACTs are effective, that timely changes to national treatment policies can be implemented, and that ART resistance can be detected early.

The mutations of the K13-propeller detected in our study mainly came from Equatorial Guinea, Angola, and other West African, South African, and Central African countries. Furthermore, the mutations corresponding to the most prevalent mutations in Cambodia were not observed in East Africa. Historically, the emergence of drug-resistant *P. falciparum* strains (to chloroquine and sulphadoxine-pyrimethamine) first occurred in Southeast Asia, spread to East Africa, and then moved outwards to the rest of the African continent [25]. But in our study, this did not seem to be so. The mutations might be due to increased international travel and migration.

In China, ACTs have been used against uncomplicated *P. falciparum* infection since 2009 [26–28]. So far no evidence has indicated that there was ART resistance in China. However, in recent years, more than 90% of the registered malaria cases were imported cases in China [29, 30]. To understand the distribution of antimalarial drug resistance is essential for the prevention, control, and elimination of malaria in China. Early surveillance of the occurrence and spreading of antimalarial drug resistance would be greatly enhanced by the application of valid antimalarial resistance molecular markers [31].

Since 2010 Henan Province has been in the malaria elimination stage, with all reports of the disease being imported and over 90% coming from migrant workers returning from Africa after 2012. Angola, Nigeria, and Equatorial Guinea were the top three countries from which the patients returned [32, 33]. In our study, 483 patients returned from 27 countries in Africa, and half of them came back from: Angola (102), Nigeria (72), and Equatorial Guinea (65). Although C580Y was not observed and the two other mutations that are associated with ART resistance were at low frequencies, this study showed that the mutated K13-propeller gene was in the migrant workers who returned from Africa, requiring our attention to the emergence of the resistance to ACTs in Africa. The use of the molecular marker K13-propeller is fundamental for surveillance in malaria control programs, in order to prolong the life span of the ACTs in Africa. Surveillance and population studies are essential for the early detection and subsequent prevention of the spread of drug resistance [34]. It is also important to change the treatment policy in a timely manner, and the information will be useful for developing and updating antimalarial guidance. Therefore, surveillance should continuously be strengthened in migrant workers returning to Henan Province, and the data may be used to enhance molecular surveillance of antimalarial resistance and informed decisions in the rational treatment policy in Henan Province.

## Conclusions

We observed 23 point mutations, including 4 synonymous mutations and 19 nonsynonymous mutations, in the K13-propeller in migrant workers who had returned from Africa. C580Y was not detected, but R539T and P574L were found in 483 samples from Africa. It was meaningful to study artemisinin resistance in returning migrant workers in Henan Province, and these data should help provide a reasonable treatment policy in Henan Province.

## Abbreviations

ACTs: Artemisinin-based Combination Therapies;
ART: Artemisinin
GMS: Greater Mekong Subregion
SNPs: Single Nucleotide Polymorphisms
WHO: World Health Organization
